# Deferiprone-induced arthropathy in thalassemia: MRI findings in a case

**DOI:** 10.4103/0971-3026.50839

**Published:** 2009-05

**Authors:** Gyan Chand, Veena Chowdhury, A Manchanda, Sapna Singh

**Affiliations:** Department of Radiodiagnosis, Maulana Azad Medical College, New Delhi-110 002, India

**Keywords:** Arthropathy, deferiprone, magnetic resonance imaging

## Abstract

Arthropathy is a well known side effect of the iron chelator deferiprone (L1); however, the imaging findings in deferiprone-induced arthropathy are not well known. In this article, we describe the typical radiographic and MRI findings in a patient receiving regular blood transfusions who developed arthropathy after long-term therapy with the oral iron chelator deferiprone (L1). Deferiprone primarily affects the articular cartilage and the changes include synovial thickening, articular cartilage thickening, and subchondral bone erosions.

Thalassemia comprises a heterogeneous group of disorders characterized by defective synthesis of α or β chains of hemoglobin. Patients with β-thalassemia major have a form of hemoglobin that is incapable of effective oxygen carriage. These patients also have severe anemia. To maintain the hemoglobin concentration and the oxygen-carrying capacity of the blood, these patients require regular blood transfusions. Although the blood transfusions increase the hemoglobin concentration, there is also excessive iron deposition in vital organs such as the heart, pancreas, liver, etc., which itself can be the cause of mortality. To circumvent this complication, iron chelation therapy is initiated in the form of deferoxamine or deferiprone (L1) (DFP). Deferoxamine is costly and has to be administered subcutaneously 4–5 days a week, which can be quite painful. Patient compliance is very poor with this drug because of these reasons and also because of side effects such as hearing loss, visual dysfunction, abnormalities of cartilage formation, and growth retardation. Due to all these reasons, over the last two decades, the oral iron chelator DFP has been increasingly used. This drug is taken orally and patient compliance is better. However, it is not entirely free of side effects, which include agranulocytosis, arthropathy, and gastrointestinal symptoms among others. We describe the MRI findings in a patient who developed arthropathy 4 years after starting DFP.

## Case Report

**Figure 1 (A, B):**
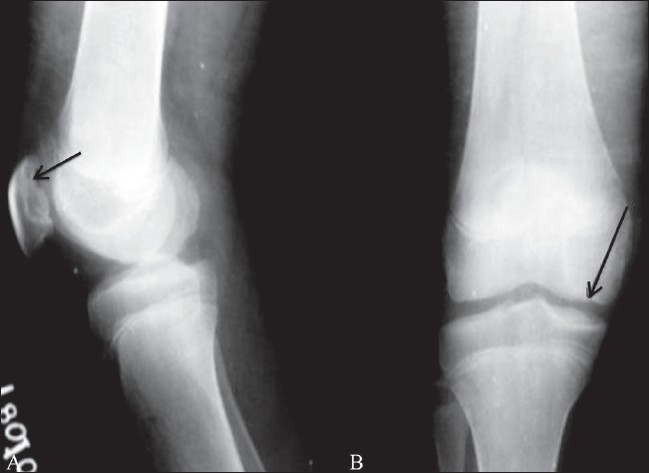
Lateral (A) and anteroposterior (B) radiographs of the left knee show patellar beaking in the upper pole and subchondral irregularity involving the articular surface (black arrow in A). Mild flattening of both the femoral condyles is seen (black arrow in B). However the metaphysis and physeal plate appear normal

**Figure 2 (A, B):**
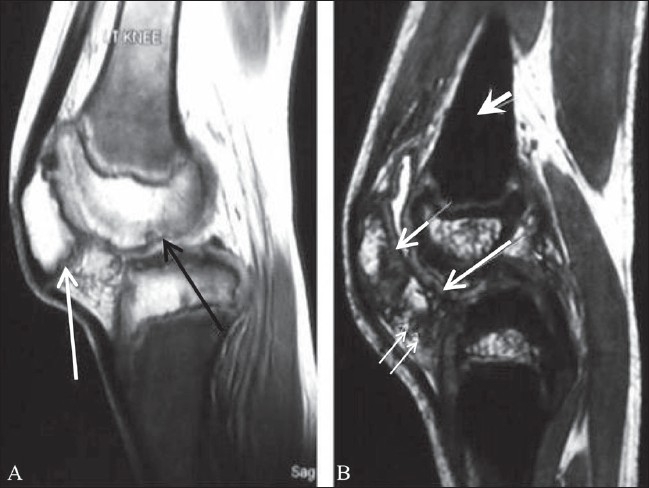
Sagittal proton-density-weighted image (A) of the left knee shows hypointense signal in the metaphyseal marrow due to hemosiderin deposition. Irregularity of the subchondral bone of the femoral condyle (black arrow) and the posterior surface of the patella (white arrow) is seen. Sagittal gradient-echo image (B) shows ‘blackout’ in the metaphyseal marrow (short thick white arrow), due to hemosiderin deposition. Marked irregular thickening of the articular cartilage of the femoral condyles (long white arrow) and patella (short white arrow) is well appreciated. There are hypointense areas seen in the infrapatellar fat pad (small white double arrows) due to hemosiderin. The synovial lining also appears black due to iron deposition

The patient is an 8-year-old boy who has been on regular blood transfusions since 3 months of age. Blood transfusion was given every 20 days and the boy was also receiving DFP 40–80 mg/kg of body weight (in accordance with serum ferritin levels) since the age of 3 years. His serum ferritin level ranged between 700–900 mg/l initially; however, he took the drug irregularly, and at times the serum ferritin levels had reached a high of 7000 mg/l. The average ferritin level was around 3000 mg/l. His total leukocyte count and neutrophil count were within normal limits. He started complaining of pain in both knees at the age of 4 years. On examination, his gait was normal and there was no swelling of the knee joints. Passive movements were within the normal range except for flexion, which was restricted. He was referred for imaging to check for evidence of arthropathy. Plain radiographs revealed mild irregular subchondral flattening of the femoral condyles [Figure 1]. Beaking of the patella was seen on the lateral view [Figure 1]. MRI of the knees showed irregular thickening of the cartilage with subchondral erosions and cartilage intrusions in these subchondral defects [Figure 2]. Joint effusion was minimal, but hypointense bands were seen in the infrapatellar fat. The marrow appeared black on all sequences due to hemosiderin deposition; this was best appreciated on gradient-echo images [dual echo in steady state (DESS)] due to susceptibility artifacts [Figure 2]. The metaphysis and the epiphyseal plate were grossly normal except for the hemosiderin deposition. Intravenous gadolinium was administered and post-contrast images did not reveal any significant abnormal synovial enhancement.

## Discussion

Because there is relatively better compliance with this drug, DFP is widely used as an iron chelator in thalassemia patients. However, it can cause various side effects, the most common being an arthropathy that usually involves the knees.[1] A few previous studies have reported that DFP-induced arthropathy is more commonly seen in patients with heavy iron loads and higher serum ferritin levels.[2,3] Aggarwal *et al*. observed that arthropathy is more commonly seen in patients on 100 mg/kg DFP and in those with a high iron load.[4]

The arthropathy or a musculoskeletal syndrome consisting of musculoskeletal stiffness, joint pain, and joint effusions may occur within a few weeks of initiation of DFP.[1] Arthroscopy reveals excess iron in the synovium and cartilage but no DFP, implying that iron may be involved in the etiology of the arthropathy.[5] Synovial biopsy performed in a few series has shown iron deposition and proliferation of the synovial lining, with no inflammatory reaction.[6] Patients suffering from transfusion siderosis associated secondary arthropathy show similar histologic findings as seen in DFP arthropathy, but the epiphyseal cartilage and subchondral bone changes are unique to DFP-related arthropathy.[3,6]

The exact etiology of the arthropathy remains uncertain, but it is hypothesized that it may be due to the toxic effects of DFP, probably mediated by free radicals and resulting from the formation of 1:1 or 1:2 DFP–iron complexes rather than the usual, inert, 1:3 complex.[1,3] No correlation was detected between the arthropathy due to DFP and antinuclear antibody, rheumatoid factor, or anti-DNA antibody.[1]

MRI findings have been described in the arthropathy due to deferoxamine;[7] however, only a few reports are available on the arthropathy due to DFP.[6] Chan *et at*. studied the MRI changes in the knees of patients taking deferoxamine. The changes included physeal–metaphyseal junctional blurring, physeal widening, distal metaphyseal lesions and pseudocystic changes, and epiphyseal and patellar lesions. However, articular changes were not seen in these cases.[7] Kellenberger *et al*. studied 14 patients receiving DFP and observed radiographic changes in 86% of cases. The changes (which they classified as mild, moderate, or severe) were joint effusion, patellar beaking, and subchondral flattening of the femoral condyles. The metaphyses were normal and the growth plates were not widened. The MRI changes they observed were synovial thickening and enhancement, synovial bands, thickening and irregular increased signal on the T2W images in the articular cartilage, and joint effusion.[6] Similar findings were seen in our case, though we did not see synovial enhancement. Most of the studies have stated that symptoms improved either with temporary discontinuation of the drug or reduction of the dose.[1] The patient in the present case report discontinued the drug for 3 months and became asymptomatic; he was later restarted on DFP. Although the imaging findings in DFP arthropathy have been reported, the long-term sequelae are not known. At present, no specific reasons can be identified for the involvement of articular and epiphyseal cartilages with DFP and the metaphyseal ossification with deferoxamine.

To conclude, the imaging features of DFP-induced arthropathy are now well recognized and the radiologist as well as the pediatrician should be aware of these. MRI is useful for assessing the synovial and cartilaginous lesions and can be of value in the follow-up of these patients.
